# Technical tips for antegrade endopancreatic radiofrequency ablation for severe pancreatojejunal stricture

**DOI:** 10.1055/a-2357-2274

**Published:** 2024-07-26

**Authors:** Takeshi Ogura, Kimi Bessho, Nobuhiro Hattori, Jun Matsuno, Hiroki Nishikawa

**Affiliations:** 1 13010 2nd Department of Internal Medicine, Osaka Medical and Pharmaceutical University, Takatsuki, Japan

Pancreatojejunal stricture (PJS) is one of the late adverse events after pancreatoduodenectomy, and can lead to pancreatitis or endocrine pancreatic insufficiency as complications 1 . PJS is usually treated under enteroscopic guidance 2 , although, because of the relatively low technical success rate and prolonged procedure time, an endoscopic ultrasound (EUS)-guided transluminal approach has recently been developed for pancreatic disease 3 . However, in cases of severe PJS, guidewire passage through the PJS into the intestine under the EUS-guided approach might be challenging, since the PJS site cannot be directly visualized. To overcome this issue, a technique involving antegrade transluminal pancreatoscope insertion has been developed. However, despite successful guidewire passage, PJS dilation might still be challenging because the pushing force might be lower in the EUS-guided approach than the enteroscopic approach. Although electrocautery dilation is a useful technique 4 , recurrence of PJS is possible since the burning effect is small. On the other hand, endobiliary radiofrequency ablation (RFA) can sufficiently burn fibrotic tissue 5 . We herein describe a novel technique for PJS treatment using RFA with a pancreatoscope.

A 77-year-old man had undergone pancreatoduodenectomy 1 year earlier for cholangiocarcinoma. At his current presentation, he was admitted to our hospital for acute pancreatitis due to PJS. First, EUS-guided pancreatic duct drainage using a plastic stent was performed. Then 2 weeks later, PJS treatment was attempted. First, guidewire passage through the PJS into the intestine was attempted, although with no success. Thereafter, a pancreatoscope (eyeMax; Micro-Tech, Nanjing, China) was antegradely inserted ( Fig. 1 ). The stricture was confirmed as being a benign tight PJS ( Fig. 2 ). Next, since the endoscopic retrograde cholangiopancreatography (ERCP) catheter could not be inserted into the intestine through the PJS site, endopancreatic RFA was attempted ( Fig. 3 ). Subsequently, the pancreatoscope was inserted and dilation of the PJS was achieved without bleeding or perforation ( Fig. 4 ). Finally, a plastic stent was deployed ( Fig. 5 ) ( Video 1 ). No recurrence of PJS or adverse events were observed at the 1-year follow-up.

**Fig. 1:**
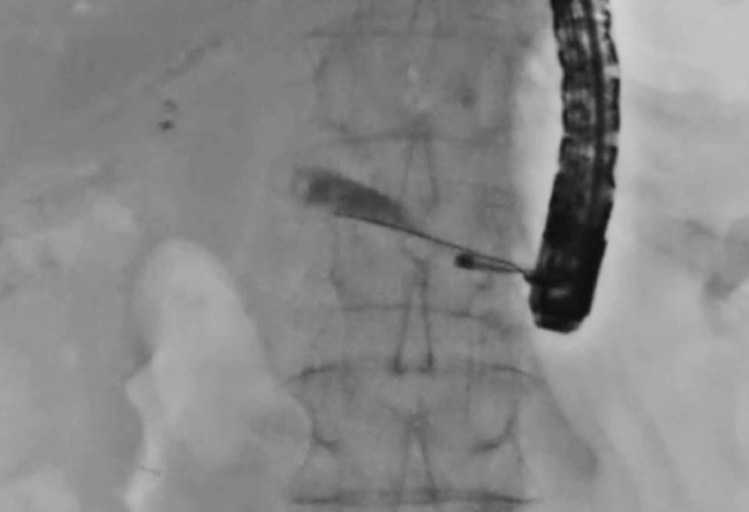
Antegrade insertion of a pancreatoscope in treatment of pancreatojejunal stricture (PJS) in a patient who had undergone pancreatoduodenectomy 1 year previously.

**Fig. 2:**
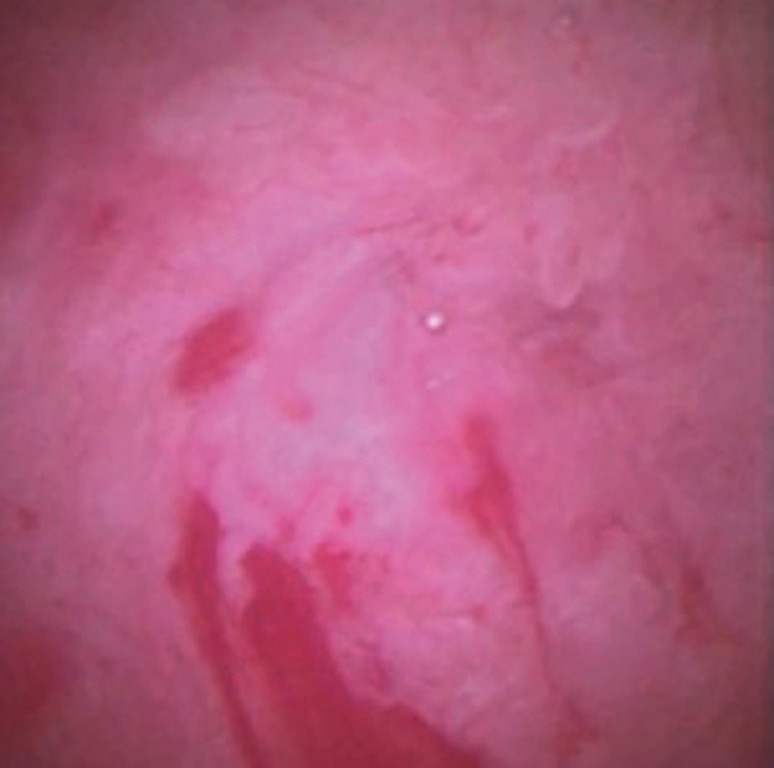
The stricture is confirmed as being a benign tight PJS.

**Fig. 3:**
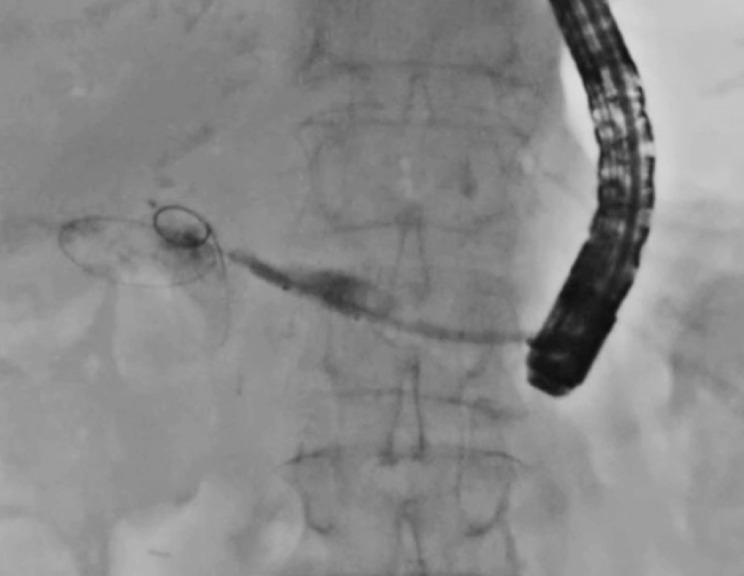
Endopancreatic radiofrequency ablation (RFA) is attempted.

**Fig. 4:**
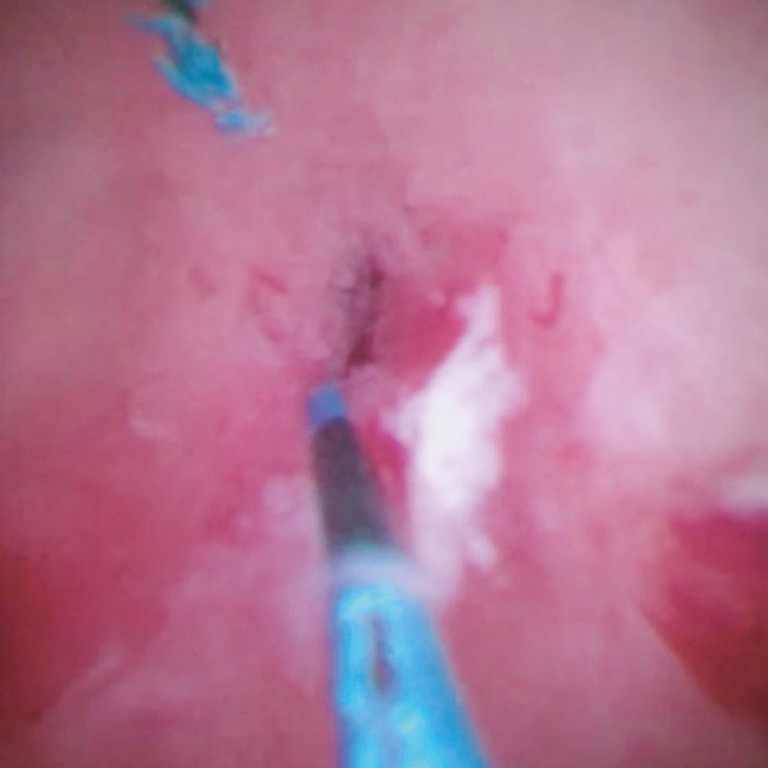
The pancreatoscope is inserted and dilation of the PJS is achieved without bleeding or perforation.

**Fig. 5:**
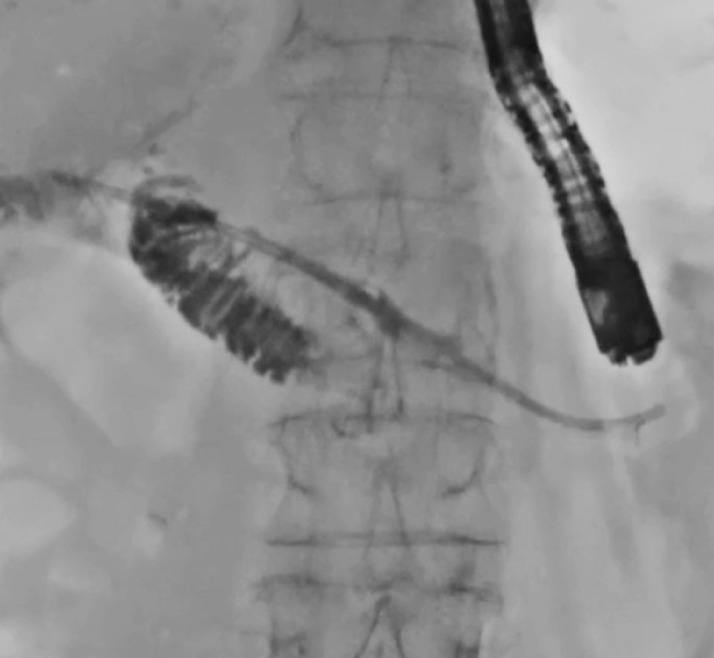
A plastic stent is deployed.

Antegrade endopancreatic radiofrequency ablation for severe pancreatojejunal stricture.Video 1

In conclusion, the presented technique might be useful for the treatment of severe PJS, although further evaluation of additional cases is required to confirm our results.

Endoscopy_UCTN_Code_TTT_1AS_2AD

## Notes

Endoscopy E-Videos https://eref.thieme.de/e-videos
*E-Videos* is an open access online section of the journal *Endoscopy* , reporting on interesting cases and new techniques in gastroenterological endoscopy. All papers include a high-quality video and are published with a Creative Commons CC-BY license. Endoscopy E-Videos qualify for HINARI discounts and waivers and eligibility is automatically checked during the submission process. We grant 100% waivers to articles whose corresponding authors are based in Group A countries and 50% waivers to those who are based in Group B countries as classified by Research4Life (see: https://www.research4life.org/access/eligibility/ ). This section has its own submission website at https://mc.manuscriptcentral.com/e-videos .
